# Evaluating LiDAR‐Derived Structural Metrics for Predicting Bee Assemblages in Managed Forests

**DOI:** 10.1002/ece3.71159

**Published:** 2025-03-27

**Authors:** Marissa H. Chase, Alexandra Harmon‐Threatt, Samuel F. Stickley, Brian Charles, Jennifer M. Fraterrigo

**Affiliations:** ^1^ Department of Natural Resources and Environmental Sciences University of Illinois Urbana Illinois USA; ^2^ Department of Entomology University of Illinois at Urbana‐Champaign Urbana Illinois USA; ^3^ Illinois Natural History Survey Champaign Illinois USA

**Keywords:** bees, forest ecology and management, LiDAR, remote sensing, structural complexity

## Abstract

Globally, many insects depend on forest habitat for critical nesting and floral resources. Forest structural complexity can affect the distribution of these resources and likewise alter insect assemblages within forests. Despite the importance of temperate deciduous forests for bees and their outsized contribution to pollination services within forests and beyond, the relationship between forest structure and bees has received scant attention. This is especially true in managed temperate deciduous forests, where management strategies alter forest structural complexity and may therefore affect bee communities. We investigated whether structural metrics derived from light detection and ranging (LiDAR) data could predict bee diversity and abundance, as well as bee functional trait composition within managed and unmanaged forests in the central hardwood region in southern Illinois, United States of America. We addressed three specific questions: (1) How does forest management affect structural complexity; (2) Can structural metrics predict bee diversity and abundance in spring and summer; and (3) How are structural metrics related to bee functional trait composition? We found that LiDAR‐derived structural metrics could not differentiate between management types and were weak predictors of bee diversity and abundance and bee functional trait composition. Metrics related to understory and midstory vegetation structure showed the strongest association with forest bee community patterns. Specifically, vegetation density in the understory (0–2 m) had a positive effect on bee diversity and abundance in spring, while in summer, vegetation density in the mid‐canopy (2–5 m) negatively affected bee communities. Our findings suggest mid‐ and understory vegetation structure, specifically vegetation density, may influence forest bee communities. Future studies should focus on the structural elements of these forest strata to improve understanding of how structural complexity influences bee communities within managed forests and evaluate the potential for using LiDAR‐derived structural metrics to monitor and predict biodiversity patterns.

## Graphical abstract

We investigated whether structural metrics derived from light detection and ranging (LiDAR) data could predict bee diversity, as well as functional trait composition within managed forest lands. Our results demonstrate that understory and mid‐story vegetation structure may have an important influence on forest bee communities. While LiDAR‐derived structural metrics were not strong predictors of bee communities, we still found that vegetation density in the understory had a positive effect on bee diversity and abundance in spring and vegetation density in the mid‐canopy had a negative effect on bee communities in the summer.

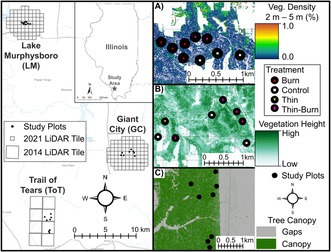

## 1 Introduction

Understanding how heterogeneity within forests drives local scale biodiversity patterns is a major research focus across many ecology and conservation disciplines (Stein et al. 2014). Forest vegetation structure is one element of heterogeneity that is a strong predictor of biodiversity (MacArthur and MacArthur 1961; MacArthur 1964; Lawton 1983). In particular, forest structural complexity (i.e., the distribution of trees and canopies in three‐dimensional space; Eckerter et al. 2021) can increase biodiversity due to increased niche space and resource diversity (Lindenmayer et al. 2000; Seidel et al. 2019). However, structural complexity has historically been difficult to quantify over extensive areas. Active remote sensing such as light detection and ranging (LiDAR) can be used to characterize structural complexity across broad spatial scales (Wulder et al. 2012; Assmann et al. 2022), but many questions remain about the usefulness of this technique for comparing structural complexity within habitat types and the extent to which these remotely sensed metrics predict biodiversity patterns (Müller and Brandl 2009; Bombi et al. 2019).

In temperate deciduous forests, management (e.g., prescribed fire and thinning) is increasingly being used to restore heterogeneous structure and composition (Arthur et al. 2012; Bragg et al. 2020). Low to moderate intensity prescribed fire can reduce mid‐story vegetation density and increase understory vegetation density, while thinning can remove vegetation density in the upper canopy and midstory and increase canopy openness (Bassett et al. 2020). However, whether these observed changes are effectively captured by LiDAR remains poorly understood, especially in forested areas with low‐intensity management. This may have particular implications for predicting shifts in insect communities, which can benefit from increases in structural complexity. For example, Knuff et al. (2020) analyzed how insect communities respond to structural complexity and found that structural complexity positively affected insect abundance due to increased niche space and resource diversity.

Temperate deciduous forests host diverse bee communities (Smith et al. 2021; Ulyshen et al. 2023; Chase et al. 2023a), with some studies showing vertical stratification of bee communities throughout the canopy (Frankie and Coville 1979; Ulyshen et al. 2010, 2020; Stangler et al. 2016; Urban‐Mead et al. 2021). Forest bee communities can be affected by thinning and burning through the promotion of floral and nesting resources caused by increased canopy openness (Romey et al. 2007; Hanula et al. 2016; Roberts et al. 2017; Ulyshen et al. 2022; Chase et al. 2023a). Given that a taxonomically and functionally diverse bee community is needed to maintain pollination services (Fontaine et al. 2006; Woodcock et al. 2019), continued bee declines (Goulson et al. 2015; Dicks et al. 2021) threaten the stability of ecosystem functioning. Improved approaches for monitoring and assessing forest structure with LiDAR data may increase capacity to predict forest bee communities and combat declining bee populations. However, knowledge of whether LiDAR‐derived structural metrics are capable of detecting forest structural characteristics that are biologically relevant to bees remains poorly understood.

A recent study by Galbraith et al. (2023) analyzing the effects of large‐scale wildfire on bee communities found that LiDAR‐derived structural metrics could be used to predict bee abundance. One other study that evaluated how forest structure and invasive shrubs affect bee diversity did not find a relationship when using LiDAR‐derived metrics (Traylor et al. 2022). However, neither study evaluated the potential of LiDAR‐derived structural metrics to predict bee functional composition (i.e., the suite of traits [e.g., nesting strategy, body size, diet breadth] that exist within a bee community) but acknowledged that bee community response to forest structure could be mediated by bee functional trait composition. Linking structure to bee functional response may be useful in evaluating how changes in bee functional trait composition affect long term pollination services. Additionally, given the limited amount of research on this subject, further studies are needed in forests that encompass a range of structural variation.

In this paper, we examined the potential of LiDAR‐derived structural metrics to predict bee diversity and abundance, as well as bee functional trait composition. Our primary goal was to improve understanding of how structural complexity affects bees within managed forests. We addressed three specific questions: (1) How does forest management affect structural complexity; (2) can LiDAR‐derived structural metrics be used to predict bee diversity and abundance in spring and summer; and (3) are LiDAR‐derived structural metrics related to bee functional trait composition? We predicted that thinning and burning would differentially affect the structure of understory and canopy layers. We expected that plots with higher overall structural complexity (i.e., increased variability of vegetation density) would have increased bee diversity and abundance because increased structural complexity would positively affect the diversity of floral and nesting resources. We also hypothesized that there would be lower bee diversity and abundance in plots with increased vegetation density because increased vegetation density would limit light availability in the understory and decrease the number of understory floral resources for bees. Additionally, we expected that structural complexity in different canopy layers would affect bee functional trait composition. We predicted that increased vegetation density and structural complexity in the upper canopy would increase cavity‐renting and cavity‐excavating bees due to increased nesting availability, while social bees would be more common in plots with increased overall vegetation density. See Table 1 for a complete list of predictions.

**TABLE 1:** Definition of LiDAR‐derived structural metrics and associated predictions.

LiDAR‐derived structural metric	Definition	Bee taxonomic response	Bee functional response
Perc_Gap	Percent canopy gap	Increased canopy gaps will be positively related to bee diversity and abundance (Romey et al. 2007; Grundel et al. 2010; Hanula et al. 2015; Hanula et al. 2016; Campbell et al. 2018; Eckerter et al. 2021)	Increased canopy gaps will be positively related to ground‐nesting bees (Vaughan et al. 2015) and negative negatively related to social and cleptoparasitic bees (Taki et al. 2013; Roberts et al. 2017; Chase et al. 2023b)
VD_MEAN	Mean overall vegetation density	Increased overall vegetation density will be negatively related to bee diversity and abundance (Romey et al. 2007; Grundel et al. 2010; Hanula et al. 2015; Hanula et al. 2016; Campbell et al. 2018; Eckerter et al. 2021)	Increased overall vegetation density will be negatively related to ground‐nesting bees (Vaughan et al. 2015) but positively related to cavity‐nesting, cleptoparasitic, and social bees (Taki et al. 2013; Roberts et al. 2017)
VD_CV	Variability of overall vegetation density (calculated as coefficient of variation)	Increased overall structural complexity will be positively related to bee diversity and abundance due to increased nesting availability (Eckerter et al. 2021)	Increased overall structural complexity will be positively related to cavity‐nesting bees due to increased nesting availability (Eckerter et al. 2021)
VH_Mean	Mean overall vegetation height	Increased vegetation height will be negatively related to bee diversity and abundance (Roberts et al. 2017; Rhoades et al. 2018)	Increased vegetation height will be positively related to social bees and negatively related to ground‐nesting bees (Roberts et al. 2017)
VH_CV	Variability of overall vegetation height (calculated as coefficient of variation)	Increased complexity of vegetation height will be positively related to bee diversity and abundance (Roberts et al. 2017)	Increased complexity of vegetation height will be positively related to cavity‐nesting bees (Roberts et al. 2017)
VD_2m_MEAN	Mean vegetation density between 0 and 2 m	Increased vegetation density between 0 and 2 m will be positively related to bee diversity and abundance due to increased floral resource availability (Campbell et al. 2007; Hanula et al. 2016; Gelles et al. 2023)	Increased vegetation density between 0 and 2 m will be positively related to oligolectic bees (Fowler 2016) and ground‐nesting bees (Hanula et al. 2016; Roberts et al. 2017)
VD_2m_CV	Variability of vegetation density between 0 and 2 m (calculated as coefficient of variation)	Increased structural complexity between 0 and 2 m will be positively related to bee diversity and abundance due to increased floral resource availability (Campbell et al. 2007; Hanula et al. 2016)	Increased structural complexity between 0 and 2 m will be positively related to oligolectic bees (Fowler 2016) and ground‐nesting bees (Hanula et al. 2016; Roberts et al. 2017)
VD_2_5m_MEAN	Mean vegetation density between 2 and 5 m	Increased vegetation density between 2 and 5 m will be negatively related to bee diversity and abundance (Hanula et al. 2015; Stangler et al. 2016)	Increased vegetation density between 2 and 5 m will be negatively related to ground‐nesting bees (Vaughn et al. 2015; Roberts et al. 2017) and positively related to cavity‐nesting and social bees (Taki et al. 2013; Roberts et al. 2017)
VD_2_5m_CV	Variability of vegetation density between 2 and 5 m (calculated as coefficient of variation)	Increased structural complexity between 2 and 5 m will be positively related to bee diversity and abundance (Ulyshen et al. 2020; Eckerter et al. 2021)	Increased structural complexity between 2 and 5 m will be positively related to cavity‐nesting bees (Eckerter et al. 2021; Urban‐Mead et al. 2021)
VD_5_15m_MEAN	Mean vegetation density between 5 and 15 m	Increased vegetation density between 5 and 15 m will be negatively related to bee diversity and abundance (Hanula et al. 2015; Stangler et al. 2016)	Increased vegetation density between 5 and 15 m will be negatively related to ground‐nesting bees (Vaughn et al. 2015; Roberts et al. 2017) and positively related to cavity‐nesting and social bees (Taki et al. 2013; Roberts et al. 2017)
VD_5_15m_CV	Variability of vegetation density between 5 and 15 m (calculated as coefficient of variation)	Increased structural complexity between 5 and 15 m will be positively related to bee diversity and abundance (Ulyshen et al. 2020; Eckerter et al. 2021)	Increased structural complexity between 5 and 15 m will be positively related to cavity‐nesting bees (Eckerter et al. 2021)
VD_Above_15m_MEAN	Mean vegetation density > 15 m	Increased vegetation density above 15 m will be negatively related to bee diversity and abundance (Romey et al. 2007; Grundel et al. 2010; Hanula et al. 2015; Hanula et al. 2016; Campbell et al. 2018; Eckerter et al. 2021)	Increased vegetation density above 15 m will be negatively related to ground‐nesting bees (Vaughn et al. 2015; Roberts et al. 2017) and positively related to cavity‐nesting, polyetic, and social bees (Roberts et al. 2017; Eckerter et al. 2021; Urban‐Mead et al. 2021)
VD_Above_15m_CV	Variability of vegetation density > 15 m (calculated as coefficient of variation)	Increased structural complexity above 15 m will be positively related to bee diversity and abundance (Ulyshen et al. 2020; Urban‐Mead et al. 2021)	Increased structural complexity above 15 m will be positively related to cavity‐nest bees (Ulyshen et al. 2010; Eckerter et al. 2021; Urban‐Mead et al. 2021

## 2 Methods

### 2.1 Study Area and Sampling Design

**FIGURE 1:**
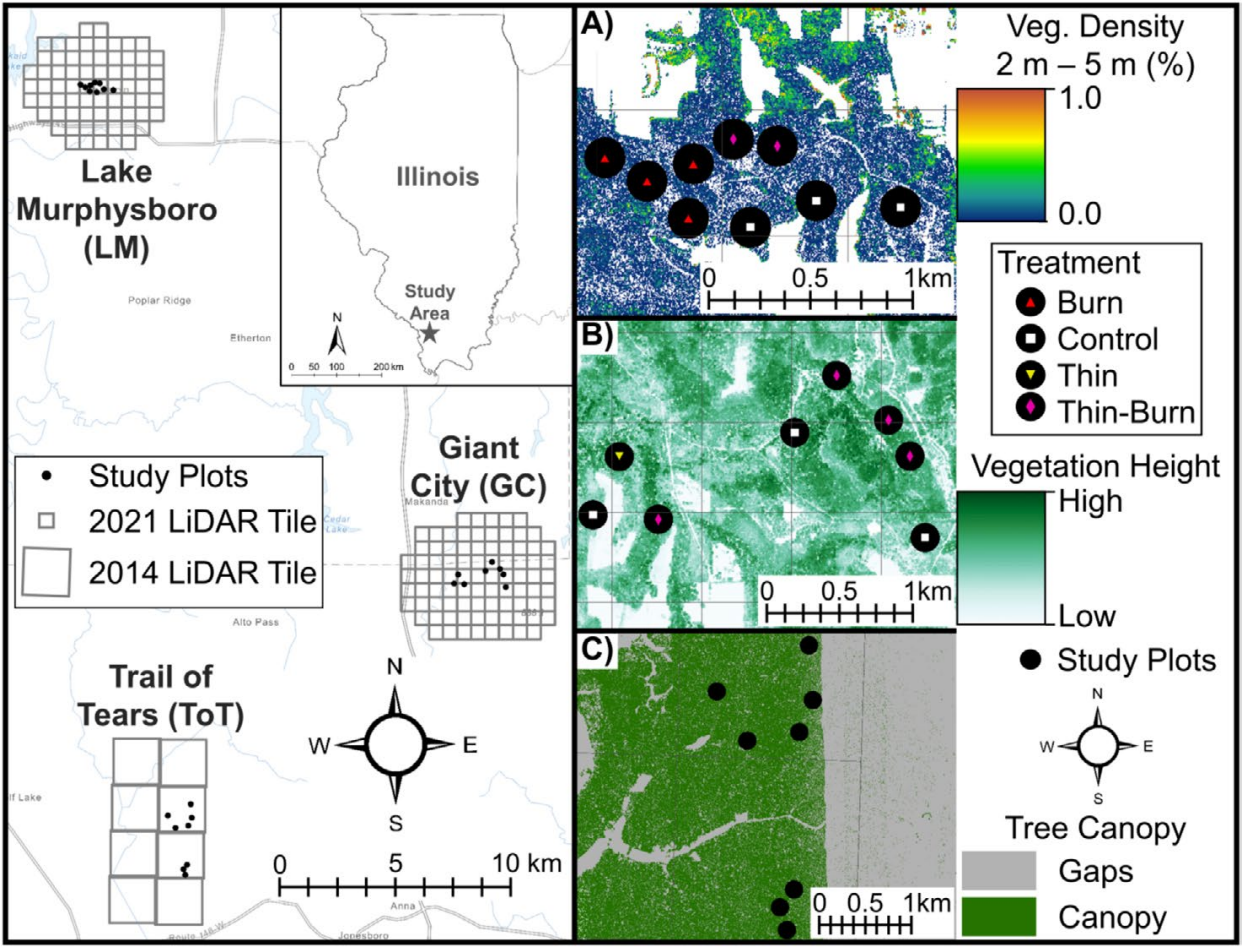
The study region in southern Illinois, USA with associated LiDAR tiles (2014 and 2021) overlayed across public land sites (left) and panels on the right demonstrating some of the important vegetation characteristics used in this study. This includes (A) LiDAR‐derived vegetation density between 2 and 5 m at the Lake Murphysboro (LM) study site, (B) LiDAR‐derived variability of forest vegetation height at the Giant City (GC) study site, and (C) percent canopy gap at the Trail of Tears (ToT) study site.

We conducted our study in southern Illinois across three different public land sites: Trail of Tears State Forest (ToT), Giant City State Park (GC), and Lake Murphysboro State Park (LM; Figure 1). Southern Illinois has a humid continental to humid subtropical climate with an average annual temperature of 13.4°C and average annual rainfall of 1198 mm from 2000 to 2020 (Midwest Region Climate Center). As this area of Illinois was unglaciated, the terrain is rugged with deeply dissected valleys and ridged uplands. These sites are broadly located within a forest‐agriculture matrix, which contains orchards and small‐scale agriculture. Deciduous, oak‐hickory forest is the dominant forest type across southern Illinois, and each public land site is similarly characterized as such with white oak ( *Quercus alba* ), northern red oak ( *Q. rubra* ), black oak ( *Q. velutina* ) and mockernut hickory ( *Carya tomentosa* ), red hickory ( *C. ovalis* ), black hickory ( *C. texana* ), and shagbark hickory ( *C. ovata* ) as dominant canopy species. Mature beech ( *Fagus grandifolia* ), sugar maple ( *Acer saccharum* ), and ironwood ( *Ostrya virginiana* ) were mixed throughout the canopy and subcanopy. Spicebush ( *Lindera benzoin* ), pawpaw ( *Asimina triloba* ), and beech saplings were some of the dominant species in the midstory and understory. In the spring, dominant herbaceous species in the understory included spring beauty ( *Claytonia virginica* ) and common blue violet ( *Viola sororia* ). In the summer, dominant herbaceous species included eastern beebalm (*Monarda bradburiana*), hoary mountain mint ( *Pycnanthemum incanum* ), Downy agrimony ( *Agrimonia pubescens* ), and Maryland figwort ( *Scrophularia marilandica* ).

We initially selected 28 100 m^2^ plots using the “Create Random Points” tool in ArcGIS v. 10.8.1 (ESRI, Redlands, CA, USA) and later ground‐truthed them. The 28 plots (ToT = 12 plots; LM = 8 plots; GC = 8 plots) were distributed across each public land site and separated by a distance of at least 300 m to ensure spatial independence. A distance of 300 m accounts for the maximum and mean foraging distances of small and medium‐large bees, respectively (Wolf and Moritz 2008; Hofmann et al. 2020), but often bees forage at much shorter distances (Harmon‐Threatt and Anderson 2023). The Illinois Department of Natural Resources (IDNR) managed these sites starting in the fall of 2014 and ending in March 2020 with four goals: restore xerophytic conditions, limit shade‐tolerant tree species, open the canopy, and restore tree diversity (see Table S1 for information regarding management specifications). Plots were divided into three treatment types based on management history—thin‐only (*N* = 4), burn‐only (*N* = 7), and thin and burn (thin + burn) (*N* = 10)—and non‐treated control plots (*N* = 7).

### 2.2 Bee and Floral Resource Sampling

To characterize bee communities and floral resources, we sampled plots twice per year in 2021 and 2022—once in early spring (late March through early April) and a second time in summer (late July through early August). Each sampling period occurred over a two‐week period to minimize seasonal turnover in the bee community. We conducted sampling on sunny days with minimal wind when temperatures were > 15.6°C.

We collected bees once during each sampling period using passive and active sampling techniques. Initially, plots were divided into four equal quadrants using perpendicular transects. Once temperatures were above the 15.6°C threshold, we placed five sets of three pan traps (blue, yellow, and white) within each plot directly on the ground. Each pan trap consisted of a solution of water and Dawn Ultra Original Scent dish soap (Droege 2012; Droege et al. 2016). Individual pan trap bowls had a diameter of 7.62 cm (3 in) at the top, 5.08 cm (2 in) at the bottom, and were 3.81 cm (1.5 in) deep. Pan trap sets were placed in the center of each quadrant, in addition to one set at the center of the plot (5 total locations), resulting in a distance of 5 m between pan trap sets. Within a set, bowls were placed roughly 23–30 cm (9–12 in) apart from each other in a triangular shape. The shape of a set and the distance within a set varied slightly depending on the terrain to keep the bowls level. After 2 h, bees were removed from the pan traps and stored in whirlpak bags.

We began active bee sampling directly after passive sampling. We hand netted bees in each plot for 20 min excluding the time required for handling bees. Each quadrant of the plot was sampled for 5 min to ensure even sampling coverage. Bees were killed with cyanide tubes, and floral association was recorded for each bee upon collection. Both netted and pan trapped bees were processed and pinned the same night. To remove excess soap, pan trapped bees were cleaned with water and 70% ethanol and blow dried at low heat. The lead author identified bees to the species level using appropriate genera keys (Gibbs 2011; LaBerge 1971, 1973; Mitchell 1960, 1962; Rehan and Sheffield 2011; Williams et al. 2014) and specimens were sent to a local taxonomist, Michael Arduser, for identification and verification.

To evaluate functional diversity, we chose four common bee functional traits that are known to respond to forest management (Chase et al. 2023b) or have been documented as bee functional traits and are related to pollination (Chase et al. 2023c: lecty, sociality, nesting strategy, and parasitism). All species were assigned a trait value by using information from the literature (see Chase et al. 2023b for specific studies). For lecty, bees were defined as either polylectic (generalist) or oligolectic (specialist). Given our species pool, sociality was divided into solitary, subsocial, or primitively eusocial. Nesting strategy was split into four different categories: ground‐excavating (ground‐nesting), cavity‐renting, cavity‐excavating, or diverse‐nesting (bee species that use multiple nesting strategies). Finally, for parasitism, bees were classified as either cleptoparasitic or non‐cleptoparasitic. Given that cleptoparasitic bees do not forage for their own resources or construct nests, we used their host's trait information for lecty and nesting strategy. Because the genus *Lasioglossum* is more variable in terms of nesting and sociality traits, we gathered trait information differently than other genera. First, we determined if there were known nesting and sociality studies for a given species. If we could find such studies, trait information was gathered using those studies. If not, trait information was assigned based on phylogenetic data within Gibbs et al. (2012). A total of three *Lasioglossum* species lacked nesting and sociality studies, so we surmised trait information from the closest ancestor of each species.

We identified all flowering planting species in the herbaceous and shrub layer to the species level. To measure floral resource availability, we counted the total number of blooms on the forest floor and within the shrub layer across each plot. Bloom totals were first recorded for each flowering species and then later pooled. Flowers were only recorded if they were open and perceived as a potential nectar or pollen source for bees. Flowers in compound umbels (e.g., some species within Apiaceae) were counted in clusters rather than counting each individual flower.

### 2.3 LiDAR‐Derived Structural Metrics

To characterize vegetation structure and structural complexity, we used LiDAR data to develop multiple structural vegetation metrics. Previous research has related canopy structural metrics to bee communities (Galbraith et al. 2023); we build upon these studies by also assessing structural metrics in the mid‐canopy and understory. Specifically, we calculated the following structural vegetation metrics across four separate strata of the forest profile: canopy segmentation (i.e., canopy cover and canopy gaps), vegetation height, and mean vegetation density and vegetation density variability (coefficient of variation (CV)) (See Table 1 for definitions of LiDAR metrics and their predicted relationships to bee communities). We downloaded LASer (LAS) files from the Illinois geospatial Data Clearinghouse (Illinois Height Modernization Program 2023) for Jackson County, IL, and Union County, IL (Figure 1). These data were acquired in 2020 for Union County and 2021 for Jackson County. Due to limited collection of above‐ground LAS points for the southern area of Union County, which encompassed plots located at Trail of Tears State Forest (ToT), we used data collected in 2014 to assess vegetation structural metrics in that region (Figure 1). Management in ToT occurred after 2014, but in Giant City (GC) and Lake Murphysboro (LM), management occurred before LiDAR data was collected in 2020 and 2021. Therefore, plots within ToT (*N* = 12) were dropped from analyses when evaluating the effects of management on vegetation structure. The vertical accuracy, as calculated by the vertical root mean square error in height meters (RMSEz(m)) for all datasets was ≤ 10 cm. The nominal pulse spacing ranged from ≤ 50 to 71 cm across all datasets. The nominal pulse density (pulse/m^2^) ranged from ≥ 2.0 for data collected in 2020 Union County and 2021 Jackson County datasets, and ≥ 4.0 for the 2014 Union County dataset.

To develop vegetation height and density metrics, we first filtered LAS points into ground and vegetation classes. We then calculated the range of height values between the lowest and highest LAS points within each cell (pixel) of a 5 m grid to estimate the tallest vegetation height per pixel. For vegetation density metrics, we also classified the LAS points into four strata across the forest profile: vegetation between 0 and 2 m (understory), vegetation between 2 and 5 m (low mid‐story), vegetation between 5 and 15 m (upper midstory), and vegetation > 15 m (canopy). The LAS points for each stratum were binned into 5 m grids to obtain the number of points per pixel. We calculated the percentage of LAS points for each stratum out of the total points (including ground) to produce a vegetation density estimate for each forest stratum at a 5 m spatial resolution. Vegetation height and density metrics were developed using LAS Dataset tools in ArcGIS v.3.0 (ESRI 2023).

We also calculated canopy cover and canopy gaps by segmenting tree canopies using the ‘lidR’ package in R version 4.1.2 (Roussel et al. 2020; Roussel and Auty 2023; R Core Team 2023). Tree segmentation was performed by locating individual tree crowns from a canopy height model, then applying a tree segmentation algorithm that rasterized the canopy at a 0.5 m spatial resolution using a 0.2 m‐radius subcircle (Dalponte and Coomes 2016). This resulted in a layer of predicted tree canopies, which we used to quantify canopy cover and gaps (percent openness) across the study area.

We summarized all structural metrics within a 100‐m buffer of each plot (Figure 1). A 100‐m buffer encompasses the potential foraging ranges of bee species and allowed us to maximize sample size without characterizing structure in overlapping buffer areas.

### 2.4 Statistical Analyses

To understand how LiDAR‐derived structural metrics varied across plots (Q1), we performed redundancy analysis (RDA) using the rda() function in the *vegan* package (Oksanen et al. 2019). RDA is a constrained ordination where one set of variables can be explained by the variation in another set of variables. Unlike principal components analysis (PCA), RDA can be used to infer how environmental conditions relate to site or species groupings (Borcard et al. 2011). Given there were limited above‐ground LAS points in the southern region of the 2021 LiDAR data, plots within Trail of Tears State Forest (ToT) (*N* = 12) were dropped from this portion of the analysis since management occurred after 2014 when the LiDAR data were collected. Permutational multivariate analysis of variance (PERMANOVA) was used to determine if structural complexity significantly differed between treatments. PERMANOVA was performed using the adonis2() function in the vegan package with 999 permutations (Oksanen et al. 2019). Next, we used the *pairwiseAdonis* package to run pairwise comparisons with 999 permutations between treatments. The amount of variance within each treatment, as calculated by the average distance to centroid, was compared across treatments using ANOVA.

We used linear models to examine the effects of LiDAR‐derived structural metrics on bee diversity and abundance in spring and summer separately (Q2). Spring and summer data were analyzed separately because a companion study in comparable sites indicated that bee composition varies temporally within temperate deciduous forests (Chase et al. 2023a). Bee diversity was calculated using Shannon's diversity index (H′) (Magurram 1988) and averaged across both sampling years. Abundance was also averaged across sampling years. Bee diversity and abundance values were log transformed to meet model assumptions of normality and homogeneity of variance. Because some of the predictor variables were on different scales (e.g., structural metrics vs. bloom abundance), we centered and scaled the predictor variables for all models to standardize coefficients and allow for comparable effect sizes. Site, which accounts for the three different public lands used in our study system (ToT, GC, and LM), was used as a fixed effect to account for unmeasured factors that were expected to change with site. We tested for collinearity between predictor variables by calculating Pearson's correlation coefficients and excluding variables from the same model if they were highly correlated (|*r*| > 0.7).

To further investigate how structural complexity affects bee community structure, we ran a series of regressions with all possible combinations of predictor variables using the dredge() function in the *MuMIN* package (Bartón 2017) and evaluated the predictive strength of models using Akaike's information criterion corrected for small sample sizes (AICc) and Akaike weights (*w*_*i*_). To prevent model oversaturation, a maximum of five parameters were allowed within each model. In addition to structural metrics and site, bloom abundance was considered as another fixed effect given its influence on bee community structure in temperate deciduous forests (Gelles et al. 2022). Models were considered competitive if they were within 2 ΔAICc units of the best‐fitting model. We then used the subset() function within *MuMIN* to calculate model‐averaged coefficients for each fixed effect and determine which variables best predicted bee diversity and abundance. Relative importance values for each fixed effect were also calculated as the sum of the Akaike weights (*w*_*i*_) over all of the supported models in which the variable of interest appears (Johnson and Omland 2004). To visualize top models for spring and summer diversity and abundance models, we plotted back‐transformed predicted values from each model with unscaled predictors using the predict() and ggplot() functions.

We used canonical analysis of principal coordinates (CAP) with the capscale() function in *vegan* to test whether LiDAR‐derived structural metrics were related to bee functional trait composition (Q3). Trait information was summed across spring and summer for both sampling years, and Bray–Curtis distances were used to calculate dissimilarity in trait composition between plots. We additionally used Procrustes analysis in *vegan* with 999 permutations to test the correlation between the principal coordinate analysis (PCoA) ordination spaces of the LiDAR‐derived structural data and bee functional trait data. Procrustes analysis scales each ordination to equal dispersions and then rotates them to maximize similarity (Peres‐Neto and Jackson 2001).

Statistical analyses were conducted with R 4.2.1 (R Core Team 2023).

## 3 Results

Across both years of sampling, we collected 1024 bee specimens belonging to 50 species and 13 genera. Six hundred fifty‐three specimens were collected in 2021 and 371 specimens in 2022. During spring, across both sampling years, we collected an average of 14.4 ± 12.9 bees in each plot. In summer, bee abundances were much lower, with an average of 3.86 ± 5.62 bees per plot. A total of 334 bees were hand‐netted, and 690 were collected with pan traps across spring and summer in both sampling years. See Table S2 for a complete list of bee species by treatment management type and collection method. Species spanned each functional trait category for nesting strategy (ground‐nesting, cavity‐renting, cavity‐excavating, and diverse‐nesting), sociality (solitary, subsocial, and primitive eusocial), lecty (oligolectic and polylectic), and parasitism (cleptoparasitic and non‐cleptoparasitic). See also Table S3 for a complete list of flowering plant species by treatment management type.

### 3.1 How Does Forest Management Affect Structural Complexity?

**FIGURE 2:**
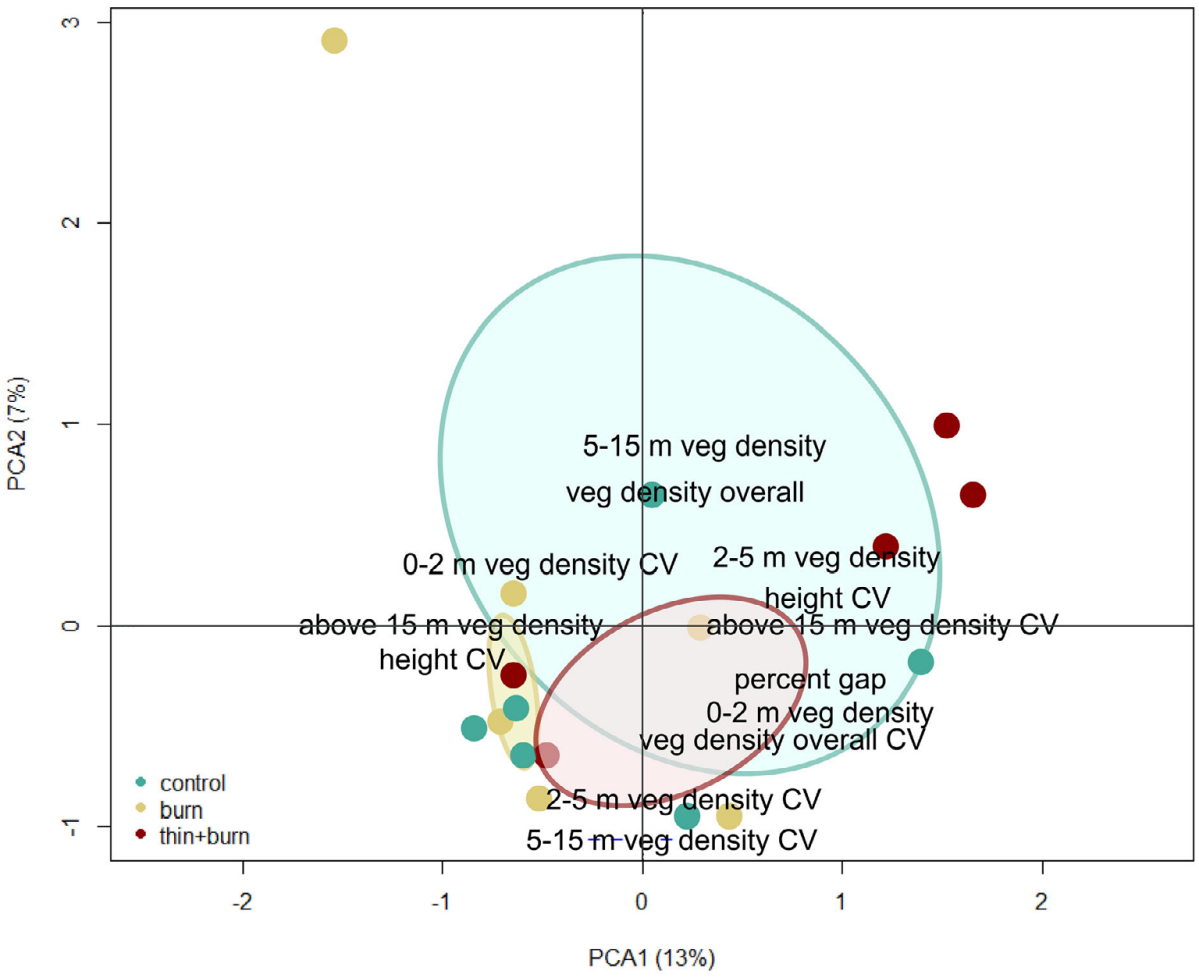
Redundancy analysis (RDA) ordination plot illustrating the relationship between LiDAR‐derived structural metrics and management type. Circles represent site scores. Strength of structural metrics are represented by distance from the origin of the plot. Percentages in axis labels indicate the amount of variation explained by each axis. LiDAR‐derived structural metrics.

Redundancy analysis (RDA) indicated LiDAR‐derived structural metrics did not significantly differ among management and control treatments (*F* = 1.25; *p* = 0.24; Figure 2). However, control plots had higher overall vegetation density, while thin + burn plots had higher variability of vegetation density between 2–5 m and 5–15 m and greater vegetation density between 0 and 2 m. Burn‐only plots had greater vegetation height in the canopy and a lower percentage of canopy gaps. The average distance to centroid was highest in control plots and lowest in burned plots, though variance was not significantly different between treatments (*F* = 2.22, *p* = 0.12).

### 3.2 Can LiDAR‐Derived Structural Metrics Be Used to Predict Bee Diversity and Abundance in Spring and Summer?

**FIGURE 3:**
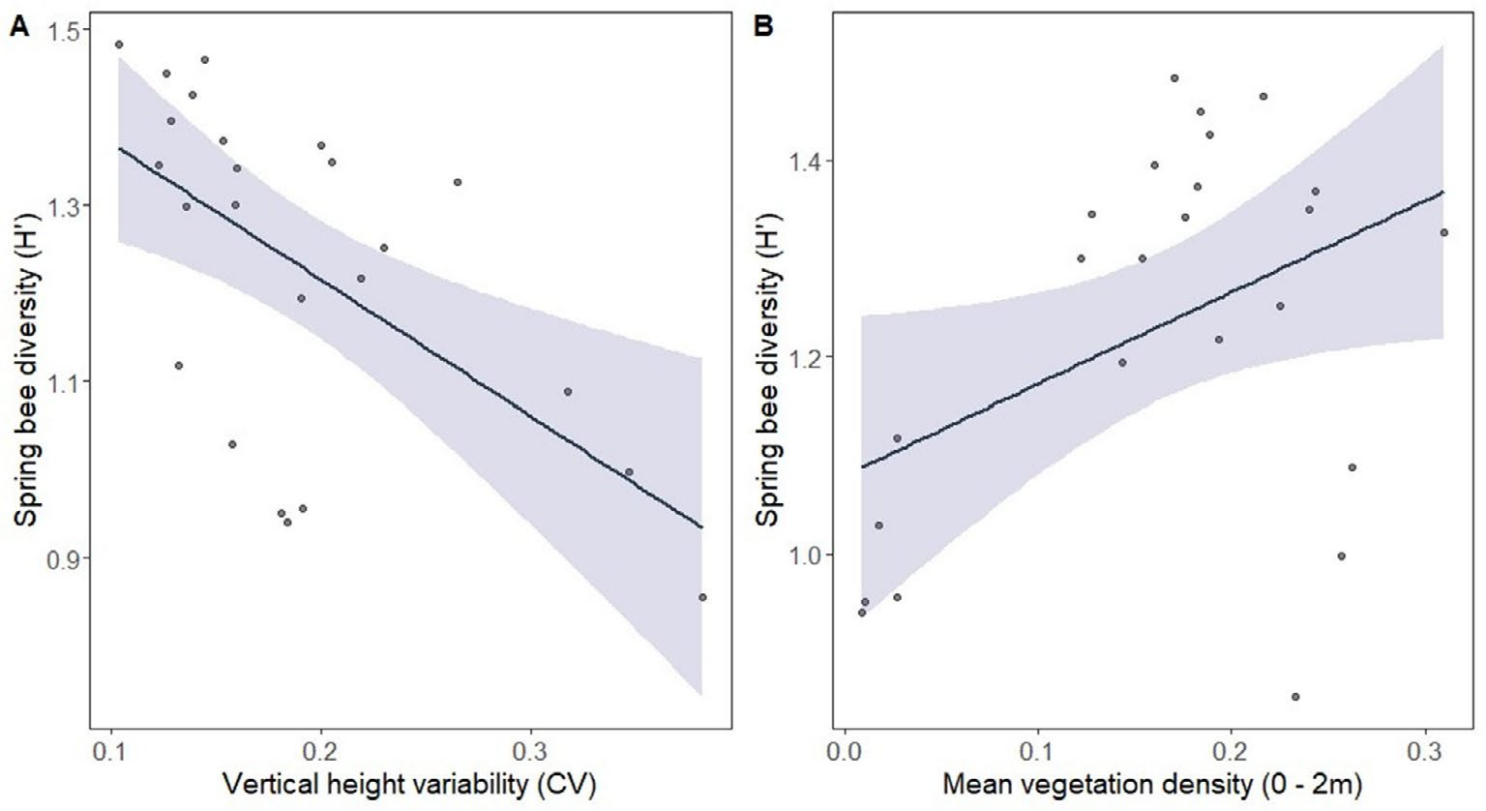
Plot depicting the results from the best‐fitting linear regression model for spring bee diversity. Relationship between vertical height variability (CV) (A) and mean vegetation between 0 and 2 m (B) and spring bee diversity. Light blue area represents the 95% confidence interval.

The most supported model of spring bee diversity included the main effects of mean vegetation density between 0–2 m and vegetation height variability (Table 2; *R* ^2^ = 0.24). These structural metrics had the highest relative importance values across all supported models (Table 3). Spring bee diversity was negatively related to overall vertical height variability (Figure 3A) and positively related to the mean vegetation density between 0 and 2 m (Figure 3B). There were three additional competing models. However, the cumulative AICc weight (*w*_*i*_) for all supported models was 0.06 (Table 2).

**TABLE 2:** AICc model summary table for spring and summer bee diversity and spring and summer bee abundance as a function of LiDAR‐derived structural metrics. Best‐fitting models (bolded) and competing models (ΔAICc < 2) are shown.

Response variables for season	Fixed effects	K	AICc	ΔAICc	AICc *w*_*i*_
Spring diversity	**0–2 veg density + height CV**	**4**	**−20.9**	**0.00**	**0.024**
	5–15 veg density	3	−19.8	1.06	0.014
	5–15 veg density + height CV	4	−19.4	1.5	0.011
	0–2 veg density CV + height CV	4	−18.9	1.99	0.009
Summer diversity	**Percent gap + site + 2–5 veg density + 0–2 veg density CV**	**7**	**4.8**	**0.00**	**0.057**
	Perc_gap + site + VD_2_5m_mean	8	5.7	0.86	0.037
Spring abundance	**Bloom abun + veg density**	**4**	**34.5**	**0.00**	**0.022**
	Bloom abun + 0–2 veg density + above 15 veg density	5	35.1	0.54	0.017
	Bloom abun + site + veg density CV	5	35.3	0.76	0.015
	Bloom abun + veg density CV + height CV	5	35.8	1.27	0.012
	Bloom abun + 0–2 veg density + above 15 veg density CV + veg density	5	35.9	1.40	0.011
	Bloom abun + 0–2 veg density + veg density	5	36.0	1.45	0.011
	Bloom abun + veg density CV	4	36.0	1.46	0.011
	Bloom abun + 0–2 veg density + veg density CV + height CV	6	36.3	1.82	0.009
	Bloom abun + 5–15 veg density CV + veg density	5	36.4	1.84	0.009
	Bloom abun + 5–15 veg density + veg density	5	36.5	1.99	0.008
Summer abundance	**Site + 2–5 veg density + 5–15 veg density + veg density CV**	**7**	**68.2**	**0.00**	**0.038**
	Percent gap + site + 2–5 veg density + 0–2 veg density CV	7	69.1	0.90	0.024
	Site + 2–5 veg density + 5–15 veg density	6	69.3	1.05	0.022

Abbreviations: abun, abundance; CV, coefficient of variation; veg, vegetation.

**TABLE 3:** Conditional averaging for bee diversity parameters. Relative importance values, estimates, standard error, and 95% confidence intervals of parameters based on AICc model averaging over all competing models (ΔAICc < 2.0) where the parameter appears. Bolded rows indicate parameters with a relative importance value > 0.6 and a 95% confidence interval that does not cross zero.

Diversity response	Parameter	Relative importance	Estimate	SE	2.5%	97.5%
Spring	Height CV	0.41	−0.07	0.07	−0.17	0.02
	0–2 veg density	0.33	0.08	0.07	−0.06	0.22
	5–15 veg density	0.33	−0.07	0.06	−0.18	0.05
	0–2 veg density CV	0.24	−0.04	0.05	−0.13	0.05
	5–15 veg density CV	0.19	0.03	0.06	−0.09	0.15
	Mean height	0.19	0.03	0.06	−0.09	0.16
	Veg density CV	0.19	−0.02	0.07	−0.16	0.12
	Percent gap	0.19	−0.03	0.06	−0.15	0.09
	2–5 veg density	0.18	−0.03	0.05	−0.14	0.08
	2–5 veg density CV	0.17	−0.004	0.07	−0.14	0.13
	Veg density	0.17	0.02	0.07	−0.12	0.15
	Above 15 veg density	0.16	−0.01	0.06	−0.13	0.11
	Bloom abun	0.16	−0.02	0.04	−0.1	0.06
	Above 15 veg density CV	0.16	−0.005	0.06	−0.13	0.12
	Site	0.07	—	—	—	—
Summer	**2–5 veg density**	**0.69**	**−0.26**	**0.11**	**−0.48**	**−0.05**
	Site	0.66	—	—	—	—
	Percent gap	0.43	−0.2	0.11	−0.43	0.03
	Above 15 veg density CV	0.31	−0.22	0.15	−0.54	0.1
	0–2 veg density CV	0.29	0.1	0.07	−0.05	0.26
	5–15 veg density CV	0.26	0.21	0.15	−0.1	0.51
	Veg density CV	0.24	−0.14	0.14	−0.42	0.14
	0–2 veg density	0.19	0.02	0.22	−0.41	0.45
	Veg density	0.18	−0.07	0.21	−0.47	0.34
	Mean height	0.17	−0.1	0.19	−0.49	0.29
	Above 15 veg density	0.17	0.11	0.13	−0.15	0.37
	5–15 veg density	0.16	0.08	0.15	−0.22	0.38
	Height CV	0.15	−0.09	0.11	−0.32	0.14
	2–5 veg density CV	0.14	0.04	0.18	−0.31	0.4
	Bloom abun	0.09	−0.02	0.07	−0.16	0.12

Abbreviations: abun, abundance; CV, coefficient of variation; veg, vegetation.

**FIGURE 4:**
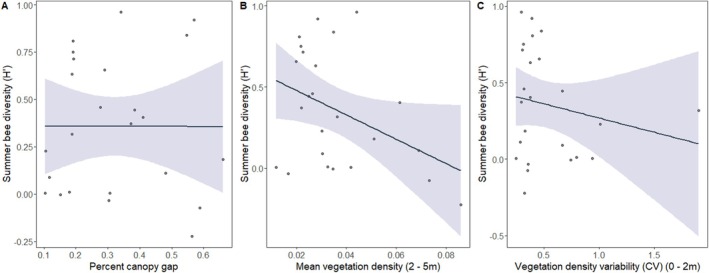
Plot depicting the results from the best‐fitting linear regression model for summer bee diversity. Relationship between percent canopy gap (A), mean vegetation between 2 and 5 m (B), and vegetation density variability between 0 and 2 m (C) and summer bee diversity. Light blue area represents the 95% confidence interval.

Contrasting spring, the most supported summer bee diversity model included the main effects of percent canopy gap, site, mean vegetation density between 2 and 5 m, and variability of vegetation density between 0 and 2 m (Table 2; *R* ^2^ = 0.62). Mean vegetation density between 2 and 5 m and site had the highest relative importance values (Table 3). Summer bee diversity was negatively related to percent canopy gap (Figure 4A), mean vegetation density between 2 and 5 m (Figure 4B), and the variability of vegetation density between 0 and 2 m (Figure 4C). There was one additional competing model, and similar to spring, combined AICc weights (*w*_*i*_) were low across both supported models (*w*_*i*_ = 0.094; Table 2).

**FIGURE 5:**
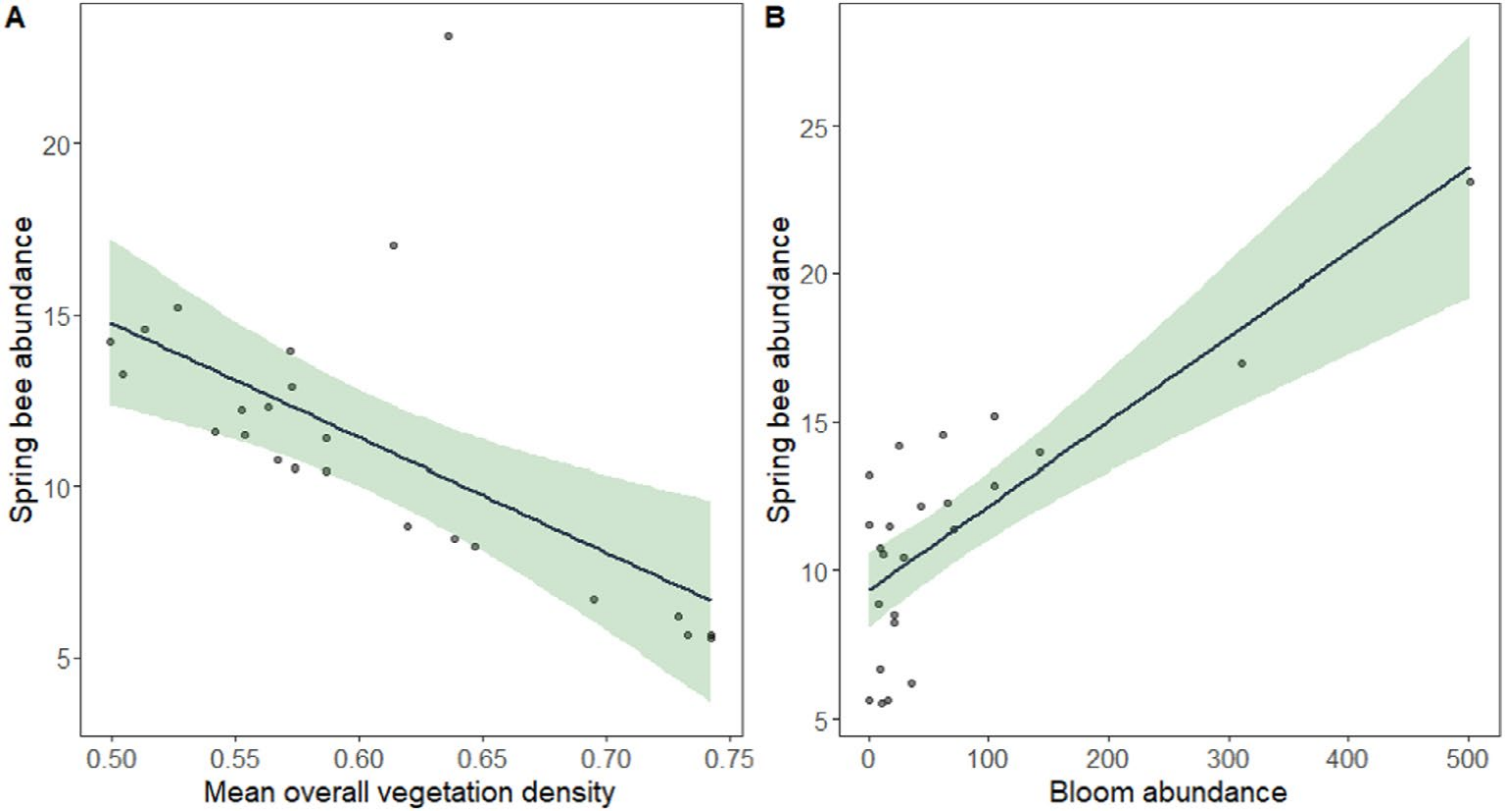
Plot depicting the results from the best‐fitting linear regression model for spring bee abundance. Relationship between mean overall vegetation density (A) and bloom abundance (B) and spring bee abundance. Light green area represents the 95% confidence interval.

Bloom abundance and mean overall vegetation density as main effects were included in the best‐fitting model of spring bee abundance (Table 2; *R* ^2^ = 0.35). Bloom abundance had the highest relative importance value (Table 4). Spring bee abundance was negatively related to mean overall vegetation density (Figure 5A) and positively related to bloom abundance (Figure 5B). There were 10 additional competing models with a combined AICc *w*_*i*_ of 0.0125 for all supported models (Table 2).

**TABLE 4:** Conditional averaging for bee abundance parameters. Relative importance values, estimates, standard error, and 95% confidence intervals of parameters based on AICc model averaging over all competing models (ΔAICc < 2.0) where the parameter appears. Bolded rows indicate parameters with a relative importance value > 0.6 and a 95% confidence interval that does not cross zero.

Abundance response	Parameter	Relative importance	Estimate	SE	2.5%	97.5%
Spring	**Bloom abun**	**0.89**	**0.28**	**0.1**	**0.07**	**0.5**
	0–2 veg density	0.35	0.29	0.21	−0.14	0.72
	Veg density	0.33	−0.21	0.17	−0.56	0.15
	Veg density CV	0.28	0.2	0.21	−0.22	0.62
	Above 15 veg density	0.22	0.15	0.2	−0.24	0.55
	Height CV	0.20	−0.15	0.18	−0.52	0.22
	5–15 veg density CV	0.19	0.15	0.18	−0.22	0.51
	Above 15 veg density CV	0.18	−0.12	0.19	−0.51	0.27
	Mean height	0.17	0.13	0.2	−0.29	0.54
	5–15 veg density	0.17	−0.05	0.25	−0.55	0.27
	Percent gap	0.16	0.12	0.18	−0.26	0.5
	0–2 veg density CV	0.15	−0.09	0.14	−0.39	0.2
	2–5 veg density CV	0.15	−0.08	0.23	−0.55	0.39
	2–5 veg density	0.14	−0.07	0.16	−0.4	0.26
	Site	0.14	—	—	—	—
Summer	2–5 veg density	0.54	−0.88	0.44	−1.77	0.01
	Site	0.51	—	—	—	—
	Above 15 veg density CV	0.37	−0.9	0.63	−2.16	0.36
	Above 15 veg density	0.34	0.68	0.5	−0.57	1.47
	Percent gap	0.29	−0.64	0.43	−1.53	0.26
	5–15 veg density CV	0.27	0.7	0.57	−0.45	1.85
	Veg density CV	0.26	−0.5	0.5	−1.51	0.51
	Mean height	0.25	−0.7	0.71	−2.11	0.72
	Veg density	0.22	0.16	0.76	−1.35	1.67
	0–2 veg density	0.2	0.3	0.8	−1.3	1.89
	0–2 veg density CV	0.19	0.34	0.31	−0.3	1.7
	5–15 veg density	0.19	0.45	0.5	−0.57	1.47
	2–5 veg density CV	0.18	0.3	0.57	−0.85	1.45
	Height CV	0.13	−0.22	0.44	−1.11	0.68
	Bloom abun	0.11	−0.15	0.23	−0.64	0.33

Abbreviations: abun, abundance; CV, coefficient of variation; veg, vegetation.

**FIGURE 6:**
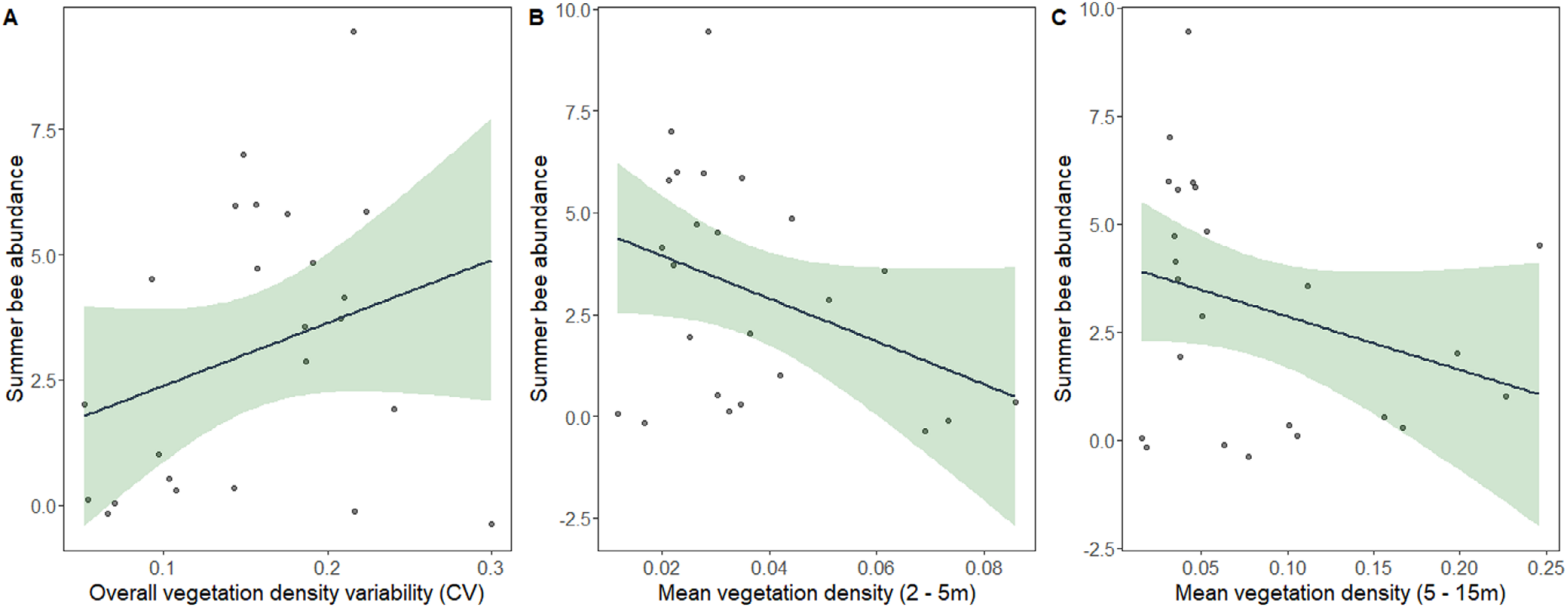
Plot depicting the results from the best‐fitting linear regression model for summer bee abundance. Relationship between overall vegetation density variability (A), mean vegetation between 2 and 5 m (B), and mean vegetation density between 5 and 15 m (C) and summer bee abundance. Light green area represents the 95% confidence interval.

Similar to the pattern of bee diversity, the best‐fitting model of summer bee abundance also differed from spring and included the main effects of mean vegetation density between 2–5 m and 5–15 m, variability of overall vegetation density, and site (Table 2; *R* ^2^ = 0.52). Mean vegetation density between 2 and 5 m had the highest relative importance value (Table 4). Summer bee abundance was positively related to the variability of overall vegetation density (Figure 6A) and negatively related to mean vegetation density between 2 and 5 m (Figure 6B) and mean vegetation density between 5 and 15 m (Figure 6C). There were two competing models; however, similar to spring bee abundance models, combined AICc *w*_*i*_ was relatively low (*w*_*i*_ = 0.088; Table 2).

### 3.3 Are LiDAR‐Derived Structural Metrics Related to Bee Functional Trait Composition?

CAP analysis indicated that bee functional trait composition was not significantly related to structural complexity (*F* = 1.5, *p* = 0.3). CAP1 and CAP2 axes explained 48% and 7% of the variation, respectively. Variability of overall vegetation height (*F* = 3.77, *p* = 0.02) and variability of vegetation density > 15 m (*F* = 6.39, *p* = 0.005) were the only significant variables. Ground‐excavating, polylectic, non‐cleptoparasitic and social bees were strongly loaded on the CAP1 axis and were negatively correlated to both significant structural metrics (Figure S1). However, Procrustes analysis indicated a low degree of concordance between structural metrics and bee functional trait PCoA ordinations (*m*_2_ = 0.89; *p* = 0.18; Procrustes rotation correlation = 0.33).

## 4 Discussion

We investigated to what extent LiDAR‐derived structural metrics could predict bee diversity and abundance, as well as bee functional trait composition within managed forest lands. Our results show that structural metrics calculated by LiDAR were weak predictors of bee diversity and abundance and bee functional trait composition. However, we found that LiDAR‐derived understory and mid‐canopy structural metrics were most important in predicting bee diversity and abundance. More specifically, vegetation density between 0 and 2 m had a positive effect on bee diversity and abundance in spring. In comparison, vegetation density between 2 and 5 m negatively affected bee communities in summer. This study provides novel information about the effectiveness of LiDAR‐derived structural metrics for predicting bee community structure and composition and the relationship between forest structure and bee biodiversity.

Contrary to our hypothesis, we did not find significant structural differences between management types; however, there were patterns associated with different treatment types. For example, thin + burn plots had greater variability of vegetation density in the mid‐canopy. This mirrors previous work that has found thin + burn treatments increase plant species richness, which can lead to changes in structural variation (Bassett et al. 2020). This is also supported by the fact that vegetation density between 0 and 2 m was greatest in thin + burn plots. Given that thinning opens the canopy and burning removes litter and regenerates nutrients, the combination of the two can have positive effects on understory plant growth (Bassett et al. 2020). However, without thinning, burning on its own may not open the canopy, which may explain why we saw greater vegetation height and smaller canopy gaps in burn‐only plots (Warner et al. 2020). The small sample size caused by excluding the southern site (Trail of Tears) from this portion of the study may have also limited our ability to detect significant structural differences between management types. Additionally, management intensity may not have been high enough to cause detectable structural differences across management categories. Prescribed fires were low–moderate surface level fires, and thinning operations only targeted mid‐canopy trees. Because of this, further research with larger sample sizes is needed to understand which LiDAR‐derived structural metrics may be most useful in detecting structural differences across management treatments.

Many studies have found that understory vegetation density is an important predictor of forest bee communities. The majority have studied this in the context of increased floral resources provided by herbaceous plants on the forest floor (Romey et al. 2007; Roberts et al. 2017; Gelles et al. 2022). In temperate forests in spring, ephemeral wildflowers such as spring beauty ( *Claytonia virginica* ), which occurred at all sites, provide some of the earliest floral resources and support a taxonomically and functionally diverse bee community (Inari et al. 2012). Our results support this, as bloom abundance outcompeted all LiDAR metrics in predicting spring bee abundance. However, increased vegetation density between 0 and 2 m also positively affected bee abundance and diversity in spring. This may reflect increased floral resources from early‐flowering woody shrubs such as spicebush ( *Lindera benzoin* ). Increased nesting availability in the form of cavities in wood may also contribute to an increase in bee abundance and diversity (Eckerter et al. 2021; Urban‐Mead et al. 2021). Given bees are central‐place foragers and nest close to floral resources (Danforth et al. 2019), increased cavity‐nesting availability in the understory may be optimal so that bees can easily access herbaceous floral resources on the ground. However, the relationship between sub‐canopy vegetation structure and bee communities is rarely evaluated.

In summer, vegetation density between 2 and 5 m had a negative effect on bee diversity and abundance. This mirrors other work that has found that subcanopy openness and not just canopy openness is a strong predictor of bee community structure (Hanula et al. 2015). This relationship is likely more apparent in summer once the canopy has leafed out (Urban‐Mead et al. 2021; Galbraith et al. 2023). The effect was especially pronounced in mesophytic forest plots, where we observed an abundance of mid‐story young beech trees and dense shade. Densely shaded stands can lead to a reduction in floral resource availability, which can have negative effects on bee communities (Grundel et al. 2010; Hanula et al. 2015, 2016; Roberts et al. 2017).

Contrary to studies that have found bee diversity and abundance are negatively associated with increased canopy cover in summer (Harrison et al. 2018), we found that the percentage of canopy gaps was negatively related to summer bee diversity. As calculated, our LiDAR‐derived percent canopy gap metric represents tree segmentation at the top of the canopy and does not necessarily reflect the openness of the canopy down to the understory. Ulyshen et al. (2010) compared bee communities in the canopy and near the ground. They concluded that bees forage in the canopy during periods of low floral resource availability to potentially acquire resins and honeydews. This may be why we found a negative relationship between percent canopy gap and summer bee diversity and abundance. As the canopy closes in summer and limits floral resources in the understory, bees may rely on additional non‐floral resources in the upper canopy (Ulyshen et al. 2010; Requier and Leonhardt 2020; Urban‐Mead et al. 2021).

Although some patterns emerged between LiDAR‐derived structural metrics and bee community structure, best‐fitting and competing models were weakly supported. This finding is in line with previous work by Traylor et al. (2022) which found that LiDAR‐derived vegetation density and vertical structural complexity did not significantly affect bee diversity and argued that not all insect groups may respond to forest structure. However, similar to our methodology, they only collected bees at one height, and different patterns may have emerged if bees were sampled at different levels of the canopy (Urban‐Mead et al. 2021; Allen and Davies 2022; Ulyshen et al. 2023). This is supported by other studies that have found bee communities can be vertically stratified throughout the canopy (Ulyshen et al. 2010; Urban‐Mead et al. 2021). Even so, Galbraith et al. (2023) sampled bees similarly to Traylor et al. (2022) and were able to detect strong relationships between LiDAR‐derived structural metrics and bee abundance (but not bee diversity). It is unclear why results from Galbraith et al. (2023) differed from our study. However, given their study occurred in a coniferous forest with high severity wildfire, results may not be comparable to our forest system.

We did not find that bee functional trait composition significantly correlated with LiDAR‐derived structural metrics. This was unexpected, as previous research has found that bee functional traits do vary with forest composition and structure in the understory (Taki et al. 2013; Roberts et al. 2017). However, these studies did not use LiDAR to assess forest structure. For example, Roberts et al. (2017) found that social bees were more prevalent in dense mature forests with fewer openings. In addition, Chase et al. (2023b) found that cleptoparasitic bees were more common in similar unmanaged stands. Perhaps what was most surprising was that cavity‐nesting bees were not associated with vegetation density in the upper canopy. This contrasts with previous work that found there were more cavity‐nesting bees in dense upper canopies because of increased nesting availability (Urban‐Mead et al. 2021). Similar to bee abundance and diversity models, the lack of significant relationships between bee functional traits and LiDAR‐derived structural metrics may stem from only collecting bees in the understory at one height level. Future research should examine the relationship between LiDAR‐derived structural metrics and bee functional trait composition at multiple levels throughout the canopy.

LiDAR metrics were not found to be strong predictors in this study, but there were some limitations with the LiDAR datasets that should be noted. For example, there was some striping noise (i.e., dark and light bands across the image) from overlapping areas in these datasets, which may have caused some minor variation in vegetation density and canopy gap averages among the study sites. However, the striping noise only affected a few study sites and did not appear to have a significant effect on our overall results. Furthermore, we had to use a LiDAR dataset from 2014 for the Trail of Tears (ToT) study area due to problems with LiDAR data collection in 2021 at this location. Therefore, the LiDAR metrics in this location should be considered conservative estimates that do not account for forest changes since that time. Given that management occurred after ToT LiDAR data were collected, these data were removed from subsequent analyses carried out to measure the effects of management on structure.

## 5 Conclusion

This is one of only three studies that have investigated whether LiDAR‐derived structural metrics can predict forest bee diversity and abundance, and the first to analyze functional trait composition in relation to structural metrics calculated from LiDAR. Although we did not find strong relationships between LiDAR‐derived structural metrics and bee diversity, bee abundance, and bee functional trait composition, our study suggests mid‐ and understory vegetation structure may have an important influence on forest bee communities. Specifically, we found that increased vegetation density in the low‐ to mid‐canopy was positively related to spring‐flying bees, and summer‐flying bees were negatively affected by increased vegetation density in the mid‐canopy. Thus, it may be useful to prioritize metrics related to these levels of the canopy in future studies. Future work should also consider assessing the size of the canopy gap, as well as the distance between gaps, to more accurately assess canopy gaps for bee communities. Additionally, future studies should consider combining LiDAR data with vegetation indices derived from passive remote sensing techniques to capture both vegetation structure and phenology. Future work could also consider other forms of LiDAR such as full waveform or more advanced LiDAR modeling approaches (e.g., voxel‐ and object‐based) to enhance vegetation metrics and structural accuracy. With increasing access to broad‐scale active remote sensing data like LiDAR (Moeslund et al. 2019), further research can evaluate the effectiveness of LiDAR data in predicting forest bee communities and potentially test different structural metrics. This is especially important in the context of managed forest lands. Management in temperate deciduous forests is increasingly being used to restore composition and structure (Arthur et al. 2012; Bragg et al. 2020) without a clear understanding of how the interaction between management and structure affects bee communities. Understanding the link between management and structure and how it impacts bee populations taxonomically and functionally will provide critical knowledge for conserving bee populations and their pollination services.

## Author Contributions

**Marissa H. Chase:** conceptualization (equal), data curation (equal), formal analysis (equal), funding acquisition (equal), investigation (lead), methodology (equal), writing – original draft (lead), writing – review and editing (lead). **Alexandra Harmon‐Threatt:** conceptualization (equal), funding acquisition (equal), methodology (equal), project administration (equal), writing – review and editing (equal). **Samuel F. Stickley:** data curation (equal), formal analysis (equal), methodology (equal), writing – review and editing (equal). **Brian Charles:** investigation (equal), writing – review and editing (equal). **Jennifer M. Fraterrigo:** conceptualization (equal), funding acquisition (equal), methodology (equal), project administration (equal), writing – review and editing (equal).

## Conflicts of Interest

The authors declare no conflicts of interest.

## Supporting information


Data S1.

## Data Availability

Data available via Dryad: https://doi.org/10.5061/dryad.j6q573nq2.
