# Supplementary material for: Evaluating LiDAR‐Derived Structural Metrics for Predicting Bee Assemblages in Managed Forests
Source: Ecol Evol. 2025 Mar 27;15(4):e71159. doi: 10.1002/ece3.71159 (PMC11949573; doi:10.1002/ece3.71159)
Supplement: Supplementary file 1 — Data S1. [file ECE3-15-e71159-s001.docx]

**Supplementary Information**

**Tables**

**Table S1:** Plot-level management information for each plot. Diameter at breast height (DBH) was measured for trees over 10 cm in diameter. Stem count was assessed for trees with a DBH of less than 10 cm.

| **Plot** | **Treatment** | **Year(s)**  **managed** | **Burn intensity** | **Basal area post-treatment** | **Stem count post-treatment** | **Target species** |
| --- | --- | --- | --- | --- | --- | --- |
| ***Trail of Tears State Forest*** | | | | | | |
| tot13 | thin-only | fall 2018 | NA | 1.57 | 67 | *Acer* spp., *Fagus grandifolia*, *Ostrya virginiana* |
| tot16 | thin-only | fall 2018 | NA | 8.21 | 47 | *Acer* spp., *Fagus grandifolia*, *Ostrya virginiana* |
| tot14 | thin-only | fall 2018 | NA | 41.25 | 42 | *Acer* spp., *Fagus grandifolia*, *Ostrya virginiana* |
| tot10 | burn-only | fall 2014; spring 2017 & 2018 | low-moderate | 19.56 | 21 | *Acer* spp., *Fagus grandifolia*, smaller understory trees |
| tot9 | burn-only | fall 2014; spring 2017 & 2018 | low-moderate | 5.67 | 29 | *Acer* spp., *Fagus grandifolia*, smaller understory trees |
| tot8 | burn-only | fall 2014; spring 2017 & 2018 | low-moderate | 8.36 | 26 | *Acer* spp., *Fagus grandifolia*, smaller understory trees |
| tot1 | thin-burn | Thin: winter 2015; Burn: spring 2018 & fall 2019 | low-moderate | 6.23 | 89 | Thin: *Acer* spp., *Fagus grandifolia*, *Ostrya virginiana*  Burn: *Liriodendron tulipifera*, *Sassafras albidum* |
| tot4 | thin-burn | Thin: winter 2015; Burn: spring 2018 & fall 2019 | low-moderate | 16.1 | 24 | Thin: *Acer* spp., *Fagus grandifolia*, *Ostrya virginiana*  Burn: *Liriodendron tulipifera*, *Sassafras albidum* |
| tot5 | thin-burn | Thin: winter 2015; Burn: spring 2018 & fall 2019 | low-moderate | 36.48 | 18 | Thin: *Acer* spp., *Fagus grandifolia*, *Ostrya virginiana*  Burn: *Liriodendron tulipifera*, *Sassafras albidum* |
| tot7 | unmanaged | NA | NA | 20.7 | 31 | NA |
| tot11 | unmanaged | NA | NA | 39.03 | 27 | NA |
| tot12 | unmanaged | NA | NA | 3.43 | 20 | NA |
| ***Giant City State Park*** | | | | | | |
| gc2 | thin-burn | Thin: winter 2019; Burn: spring 2016 & 2020 | low-moderate | 0 | 58 | Thin: 1-8” DBH;  Burn: *Ostrya virginiana*, *Fraxinus* spp., *Acer rubrum*, *Liquidambar styraciflua*, *Ulmus* spp., *Liriodendron tulipifera* |
| gc3 | thin-burn | Thin: winter 2019; Burn: spring 2016 & 2020 | low-moderate | 16.43 | 32 | Thin: 1-8” DBH;  Burn: *Ostrya virginiana*, *Fraxinus* spp., *Acer rubrum*, *Liquidambar styraciflua*, *Ulmus* spp., *Liriodendron tulipifera* |
| gc4 | thin-burn | Thin: winter 2019; Burn: spring 2016 & 2020 | low-moderate | 11.21 | 23 | Thin: 1-8” DBH;  Burn: *Ostrya virginiana*, *Fraxinus* spp., *Acer rubrum*, *Liquidambar styraciflua*, *Ulmus* spp., *Liriodendron tulipifera* |
| gc7 | thin-burn | Thin: winter 2019; Burn: spring 2017 & winter 2021 | low-moderate | 9.87 | 36 | Thin: 1-8” DBH;  Burn: *Ostrya virginiana*, *Fraxinus* spp., *Acer rubrum*, *Liquidambar styraciflua*, *Ulmus* spp., *Liriodendron tulipifera* |
| gc6 | thin-only | winter 2019 | NA | 8.67 | 5 | Thin: 1-8” DBH |
| gc1 | unmanaged | NA | NA | .87 | 19 | NA |
| gc8 | unmanaged | NA | NA | 4.01 | 21 | NA |
| gc5 | unmanaged | NA | NA | 2.64 | 11 | NA |
| ***Lake Murphysboro State Park*** | | | | | | |
| lm1 | thin-burn | Thin: spring 2019; Burn: winter 2020 | low-moderate | 17.19 | 10 | 1-8” DBH |
| lm2 | thin-burn | Thin: spring 2019; Burn: winter 2020 | low-moderate | 28.36 | 14 | 1-8” DBH |
| lm4 | burn-only | winter 2020 | low-moderate | 10.52 | 22 | 1-8” DBH |
| lm6 | burn-only | winter 2020 | low-moderate | 13.76 | 6 | 1-8” DBH |
| lm7 | burn-only | winter 2020 | low-moderate | 7.29 | 4 | 1-8” DBH |
| lm3 | burn-only | winter 2020 | low-moderate | 5.65 | 41 | 1-8” DBH |
| lm5 | unmanaged | NA | NA | 54.28 | 43 | NA |
| lm8 | unmanaged | NA | NA | 25.48 | 23 | NA |

**Table S2:** Bee species caught within each treatment type. An asterisk represents any species that was only caught passively with pan traps.

|  | **Bee family** | | | | |
| --- | --- | --- | --- | --- | --- |
|  | **Andrenidae** | **Halictidae** | **Megachilidae** | **Colletidae** | **Apidae** |
| **Control** | *Andrena imitatrix, A. violae*, A. erigeniae, A. rugosa, A. cressonii, A. nasonii* | *Augochlora pura, Lasioglossum zephryrus, L. gotham, L. cressonii, L. hitchensi, L. versatum, L. birkmanni, L. weemsi, L. (Dialictus) sp. 2** | *Osmia taurus, O. bucephala*,*  *O. atriventris*, O. pumila,* | *Colletes inequalis* | *Nomada pygmaea, N. depressa, N. luteoloides, N. armatella, N. (Gnathathis) sp. 1*, Ceratina calcarata** |
| **Thin** | *Andrena imitatrix, A. violae*, A. erigeniae, A. cressonii, A. carlini, A. nasonii* | *Lasioglossum versatum, L. weemsi, L. cressonii, L. hitchensi, Augochlorella aurata, Augochlora pura, Sphecodes heraclei* | *Osmia taurus, O. atriventris** |  | *Nomada pygmaea, N. luteoloides* |
| **Burn** | *Andrena imitatrix, A. erigeniae, A. rugosa, A. violae*, A. nasonii, A. cressonii, A. mandibularis** | *Lasioglossum cressonii, L. hitchensi, L. versatum, L. weemsi, L. birkmanni, L. gotham, L. coeruleum, L. fuscipenne, L. tegulare*, L. (Dialictus) sp. 3, L. (Dialictus) sp. 1*, Augochlora pura, Augochlorella aurata, Halictus parallelus*, Augochloropsis metallica* | *Osmia pumila, O. taurus, Megachile mendica* | *Colletes inequalis* | *Nomada pygmaea, N. armatella, N. sayi, N. luteoloides, N. sayi, N. (Nomada) sp. 1*, N. (Nomada) sp. 2*, N. salicis*, N. denticulata*, Ceratina calcarata** |
| **Thin+burn** | *Andrena imitatrix, A. violae*, A. erigeniae, A. carlini, A. cressonii, A. rugosa, A. barbara, A. nasonii, A. forbesii* | *Lasioglossum cressonii, L. weemsi, L. gotham, L. leviense*, L. versatum, L. hitchensi, L. zephyrus, L. (Dialictus) sp. 1, L. (Dialictus) sp. 2*, Augochlorella aurata, Augochlora pura, Augochloropsis metallica* | *Osmia atriventris*, O. pumila* | *Hylaeus illinoisensis, Colletes inequalis* | *Nomada pygmaea, N. armatella, N. luteoloides, N. salicis*, N. depressa*, *Ceratina strenua, C. calcarata*, Bombus impatiens* |

**Table S3.** Flowering plant species by treatment type.

|  | **Flowering plant species by treatment** |
| --- | --- |
| **Burn** | \| *Ageratina altissima* \| \| --- \| \| *Agrimonia rostellata* \| \| *Antenoron virginianum* \| \| *Circaea lutetiana* \| \| *Claytonia virginica* \| \| *Desmodium nudiflorum* \| \| *Hackelia virginiana* \| \| *Hylodesmum pauciflorum* \| \| *Phryma leptostachya* \| \| *Scrophularia marilandica* \| \| *Scutellaria incana* \| \| *Solanum ptychanthum* \| \| *Verbena urticafolia* \| \| *Viola sororia* \| |
| **Thin+burn** | \| *Agrimonia rostellata* \| \| --- \| \| *Claytonia virginica* \| \| *Desmodium nudiflorum* \| \| *Erigeron annuus* \| \| *Geum canadense* \| \| *Hackelia virginiana* \| \| *Helianthus strumosus* \| \| *Hieracium gronovii* \| \| *Hylodesmum pauciflorum* \| \| *Lobelia inflata* \| \| *Oxalis stricta* \| \| *Phryma leptostachya* \| \| *Pycnanthemum incanum* \| \| *Pycnanthemum virginianum* \| \| *Rhus aromatica* \| \| *Scrophularia marilandica* \| \| *Scutellaria incana* \| \| *Viola sororia* \| |
| **Thin** | \| *Claytonia virginica* \| \| --- \| \| *Desmodium nudiflorum* \| \| *Lindera benzoin* \| \| *Persicaria punctata* \| \| *Phryma leptostachya* \| \| *Tipularia discolor* \| \| *Viola sororia* \| |
| **Control** | *Agrimonia rostellata*  *Alliaria petiolaria*  *Antenoron virginianum*  *Cardamine concatenata*  *Claytonia virginica*  *Corydalis flavula*  *Desmodium nudiflorum*  *Geum canadense*  *Phytolacca americana*  *Sibara virginica*  *Stellaria media*  *Verbena urticafolia*  *Viola sororia* |

**Figures**

**
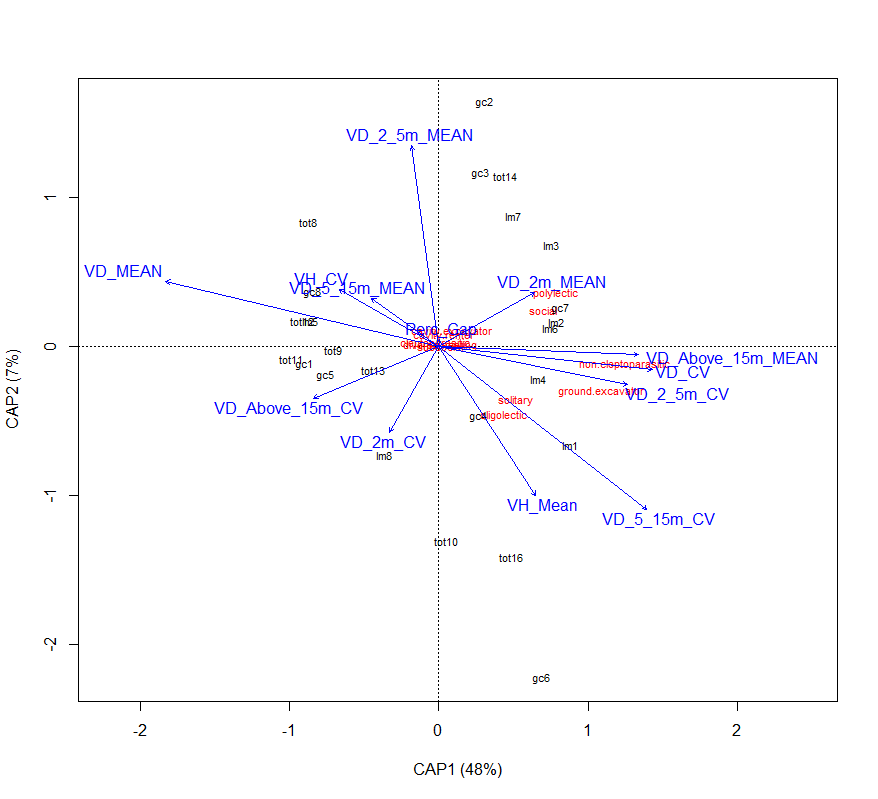
**

**Figure S1.** Canonical analysis of principal coordinates (CAP) ordination plot illustrating the relationship between bee functional trait abundances and LiDAR-derived structural metrics. Site scores are plotted as text labels. Percentages in axis labels indicate the amount of variation explained by each axis. LiDAR-derived structural metrics: VD_Above_15m_MEAN = mean vegetation density > 15 m; VH_Mean = mean overall vegetation height; VD_5_15m_CV = variability of vegetation density between 5 - 15 m; VD_2_5m_CV = variability of vegetation density between 2 - 5 m; VD_CV = variability of overall vegetation density; VD_2m_MEAN = mean vegetation density between 0 - 2 m; Perc_Gap = percent canopy gap; VD_Above_15m_CV = variability of vegetation density > 15 m; VH_CV = variability of overall vegetation height; VD_2_5m_MEAN = mean vegetation density between 2 - 5 m; VD_5_15m_MEAN = mean vegetation density between 5 - 15 m; VD_MEAN = mean overall vegetation density; VD_2m_CV = variability of vegetation density between 0 - 2m.
